# Use of Pharmacological and Non-Pharmacological Strategies by Community-Dwelling Adults to Manage Migraine: A Systematic Review

**DOI:** 10.3390/clinpract13030051

**Published:** 2023-04-23

**Authors:** Srujitha Marupuru, Ziyad Almatruk, Marion K. Slack, David R. Axon

**Affiliations:** 1 Department of Pharmacy Practice and Science, R. Ken Coit College of Pharmacy, The University of Arizona, 1295 N. Martin Ave., Tucson, AZ 85721, USA; 2 Department of Pharmacy Practice, Faculty of Pharmacy, King Abdulaziz University, P.O. Box 80200, Jeddah 21589, Saudi Arabia; 3 Center for Health Outcomes and PharmacoEconomic Research (HOPE Center), R. Ken Coit College of Pharmacy, The University of Arizona, 1295 N. Martin Ave., Tucson, AZ 85721, USA

**Keywords:** migraine, systematic review, pain management, community-dwelling adults

## Abstract

Migraine is a prevalent disease associated with high levels of disability and is often underdiagnosed and undertreated. This systematic literature review aimed to identify the types of pharmacological and non-pharmacological strategies that community-dwelling adults report using to manage migraine. A systematic literature review of relevant databases, grey literature, websites, and journals was conducted from 1 January 1989 to 21 December 2021. Study selection, data extraction, and risk of bias assessment were completed independently by multiple reviewers. Data were extracted on migraine management strategies and categorized as opioid and non-opioid medications and medical, physical, psychological, or self-initiated strategies. A total of 20 studies were included. The sample sizes ranged from 138 to 46,941, with a mean age of 34.7 to 79.9 years. The data were typically collected using self-administered questionnaires (nine studies), interviews (five studies), online surveys (three studies), paper-based surveys (two studies), and a retrospective database (one study). Community-dwelling adults with migraine reported they primarily used medications, specifically triptans (range 9–73%) and non-steroidal anti-inflammatory drugs (NSAIDs) (range 13–85%) to manage migraine. Except for medical strategies, the use of other non-pharmacological strategies was low. Common non-pharmacological strategies included consulting physicians (range 14–79%) and heat or cold therapy (35%).

## 1. Introduction

Pain is a global problem that affects many people. Pain is the leading cause of disability and one of the most common reasons to seek health care, affecting more than 30% of the population worldwide [1]. One major type of pain that often limits people’s daily activities and significantly impacts their overall quality of life is migraine. The exact cause of migraine is unknown, but it is defined as a chronic neurological disorder characterized by recurrent episodes of headache and debilitating symptoms [2]. Symptoms in adults vary based on the phases of migraine attacks; the most frequent symptoms are mood changes, vision, and sound sensitivity, followed by headache, nausea, and exhaustion [3]. Globally, it is estimated that migraine affects around 10% of people. Migraine is more frequent among people aged 20–50 years and is about three times more prevalent in women than in men [2,4]. In the United States (US), the prevalence of migraine is 18% in women and 6% in men, and it is most common between the ages of 18 and 44 [5]. Migraine imposes a substantial economic burden on families as healthcare costs are 70% higher for a family with a migraineur than for a non-affected family [6]. Migraine is a chronic disease that has a significant burden on people, families, and society. According to a study using data from the Global Burden of Disease, it was projected that migraines were responsible for 45.1 million years lived with disabilities in 2016 [7].

Despite being a prevalent disease with incapacitating symptoms, migraine remains a poorly understood disorder that is often undiagnosed and undertreated [8]. Only about 48% of migraineurs who met the clinical definition of migraine have been diagnosed by a physician [9]. Furthermore, there were only about 564 certified headache specialists in the US for over 47 million migraineurs [10]. This shortage of headache specialists results in high self-medication overuse in terms of analgesics due to easy direct access to medication. Self-management is commonly used in migraine conditions alongside pharmacological interventions, particularly when other treatment options have failed or due to non-pharmacological preference by the patient [11]. The 2007 American Migraine Study II reported that around 57% of migraineurs use over-the-counter (OTC) medications despite advanced prescription medications [12]. Although there are a wide variety of non-pharmacological strategies available for migraine self-management, including physical strategies such as yoga, psychological strategies such as meditation, and herbal remedies, no studies have comprehensively synthesized the different types of management strategies that are used [13]. Awareness of non-pharmacological therapy as an adjunct or alternative to drug treatment when it is not effective or poorly tolerated may lead to more holistic and higher-quality care. A greater understanding of how people self-manage migraine could enable healthcare professionals such as physicians and pharmacists to improve their collaboration with patients, optimize drug therapies, and increase patients’ ability to reduce the influence of migraine on their lives. Therefore, the primary objective of this study was to identify the types of pharmacological and non-pharmacological strategies that community-dwelling adults report using to manage migraine using a systematic literature review. The secondary objective of this study was to discuss the implications of these findings for clinical practice.

## 2. Methods

### 2.1. Eligibility Criteria

To be included in this systematic literature review, studies must have included community-dwelling adults aged 18 to 80 years who had migraine and who self-reported the types of strategies they used to manage their migraine. The report needed to be written in English and published after 1989. Studies that did not differentiate migraine from other types of headaches, studies that only involved migraine prevention, and studies that did not include self-reported data on strategies used were excluded. Finally, to avoid studies published in questionable (or predatory) journals, studies had to be indexed in PubMed, *Directory of Open Access Journals (DOAJ)*, or be associated with a professional or healthcare organization.

### 2.2. Search Procedure

The terms “migraine”, “self-management”, “community adults”, and “self-reported” were used to develop the search. A combination of these keywords and controlled vocabulary were utilized to develop the search strategies to identify studies in the following bibliographic databases: *PubMed/Medline (National Library of Medicine)*, *Embase (Elsevier)*, *PsycINFO*, *AMED*, *Global Index Medicus*, *International Pharmaceutical Abstract (IPA)*, *Web of Science (Clarivate Analytics)*, *Academic Search Ultimate and Scopus (Elsevier)*. Additionally, the periodical, *Journal of Headache*, *Journal of Pain*, was searched, and the reference lists of identified studies. The grey literature search included Open Grey, OAIster, Dissertations and Theses, Grey Literature Report, and websites such as the American Pain Society, Centers for Disease Control and Prevention (CDC), and Google. In addition, citation searches of identified studies were conducted in Scopus. The search strategy developed in PubMed/Medline was used to translate to the other bibliographic databases. The search dates were from January 1990 to December 2021. The search strategy is presented in Appendix A.

### 2.3. Primary Variables of Interest

The primary variable of interest was the type of pharmacological and non-pharmacological strategies used to manage migraine. To facilitate categorization, the types of strategies were divided into domains [13]. The medication domain included pharmacological strategies such as triptans, ergotamine, opioids, non-steroidal anti-inflammatory drugs (NSAIDs), paracetamol, aspirin, unspecified analgesics, analgesic and caffeine, sedatives, steroids, antinausea, OTC, and prescribed medications. Non-pharmacological strategies were divided into the domains of medical strategies, physical strategies, psychological strategies, and self-initiated strategies. Examples of medical strategies could include consulting a general physician, a specialist physician, and emergency department visits. Examples of physical strategies could include massage, acupuncture, hot and cold modalities, and exercise. Examples of psychological strategies could include relaxation, yoga, rest, and psychotherapy. Examples of self-initiated strategies could include using herbal products, homeopathy/naturopathy, diet change, and dietary supplements to manage migraine. Data were also collected on any other reported outcomes, for example, treatment efficacy and satisfaction.

### 2.4. Screening Procedure

Two independent investigators (S.M. and Z.A.) screened each record for eligibility using a standardized tool developed specifically for this study. The screening tool was designed to identify if the record contained relevant data for: (1) study characteristics, such as the study design and the source of the population; (2) patient characteristics, such as the number of participants and gender; and (3) management strategies, such as the medication used and the level of satisfaction. In cases where it was unclear if a study should be included or not, it was included for full-text review.

### 2.5. Data Extraction Tool

A standardized data extraction tool was used to obtain data from the included studies. Data were collected on any type of strategy that study participants reported using to manage their migraines. Data were also collected on study characteristics, including the country where the study was conducted, the purpose of the study, mean age of participants, the total number of participants, percentage female participants, source population, and type of survey (i.e., telephone, self-administered) used to collect the data. Data were extracted independently by two investigators (S.M. and Z.A.), and any differences were resolved by consensus through discussion with the rest of the research team (M.K.S. and D.R.A.).

### 2.6. Risk of Bias Assessment

A tool was specifically developed for this study to assess the risk of bias for identifying types of strategies used to manage migraine. Studies were assessed for: purpose (i.e., was the primary purpose of the study to identify migraine management strategies), survey reliability (i.e., to ensure the integrity and quality of survey responses since we were interested in self-reported migraine management strategies), data collection methods used (i.e., to understand methods of data collection such as telephonic, paper-based or computer-based and if interviewers were trained for the purpose), sample size (i.e., a very small sample size would reduce the likelihood of identifying a variety of management strategies) and conflict of interest (i.e., evaluate if the investigators had a stake in the success of the study). Additionally, examined were the domains used to describe the types of strategies reported because a small number of domains would limit the types of strategies identified. Each domain was scored as low, moderate, high, or unclear risk of bias.

### 2.7. Data Analysis

The extracted data were organized using an adapted version of the conceptual model developed by Axon et al. [13]. This conceptual model categorizes pain management strategies as pharmacological or non-pharmacological and further categorizes pharmacological strategies as prescription or non-prescription and non-pharmacological strategies as medical procedures, physical therapies, psychological approaches, and self-initiated strategies. The data on the management strategies reported were categorized by domain and then summarized in a table as opioid medications, non-opioid medications, physical, medical, psychological, or self-initiated strategies, as well as outcomes. The percentage of respondents using each type of strategy for each study was reported.

## 3. Results

### 3.1. Study Selection

**Figure 1:**
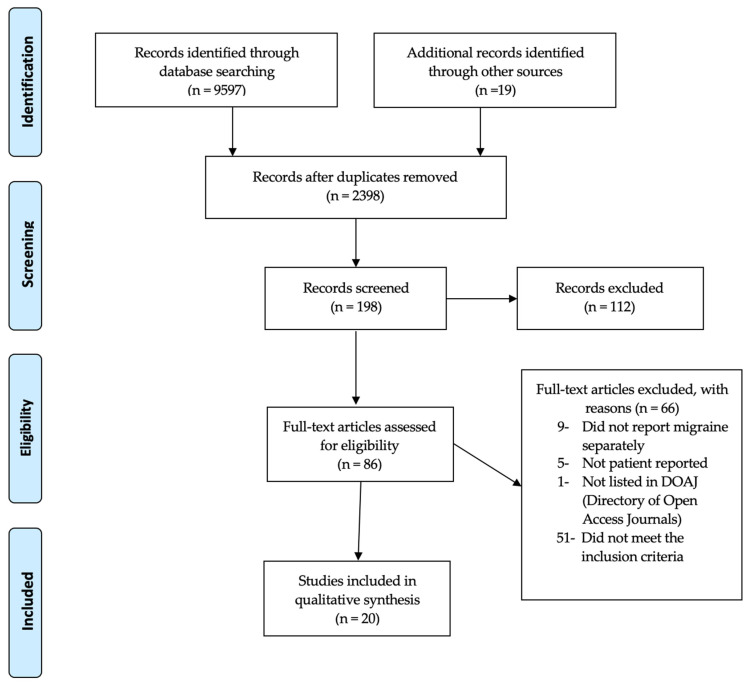
PRISMA flow chart.

As shown in Figure 1, a total of 2398 unique records were identified and screened. Of these, 86 studies underwent full-text review to further determine their eligibility for inclusion. A total of 66 of these articles did not meet our inclusion criteria and were excluded from the analysis. A total of 20 articles were ultimately included in this systematic review [14,15,16,17,18,19,20,21,22,23,24,25,26,27,28,29,30,31,32,33].

### 3.2. Study Characteristics

The characteristics of the 20 included studies [14,15,16,17,18,19,20,21,22,23,24,25,26,27,28,29,30,31,32,33] from 12 different countries are reported in Table 1. The number of subjects in each study ranged from 100 [23] to 17,071 [24]. Seventeen studies reported the percentage of females [15,16,18,19,20,21,23,24,25,26,27,28,29,30,31,32,33], which ranged from 23% [21] to 100% [18] of subjects. Sixteen studies reported the average age [15,16,17,20,21,23,24,25,26,27,28,29,30,31,32,33], which ranged from 31.7 [28] to 50.1 years [21].

The included studies had diverse source populations. Twelve studies were in the general adult population in the US [14,29], Taiwan [17], Canada [18,22], Turkey [23], Japan [24], Russia [28], France [30], United Kingdom [31], Italy [32], and Croatia [33]. Three studies involved healthcare professionals (physicians, pharmacists, and neurologists) in France [19,20,21]. Two studies involved pharmacy customers in Italy [15,16]. One study involved headache clinic patients in Kuwait [25], another study identified respondents through a headache registry in Canada [26], and one study used an interactive chronic illness panel [27] in the US.

In terms of data source, three studies conducted telephone interviews [14,18,22], nine studies reported results from self-administered questionnaires [15,19,20,21,27,30,31,32,33], two studies were paper-based questionnaires [16,23], two studies were face-to-face interviews [17,28], three studies were online surveys [24,25,29], and the study was a retrospective database study [26].

### 3.3. Types of Pharmacological Strategies Reported

As shown in Table 2, the most frequently reported pharmacological strategies used for managing acute migraine appeared to be triptans, ergotamine, and NSAIDs. Triptans were reported in 15 studies (range of reported use = 6 to 73%) [15,16,18,19,20,21,24,26,27,28,29,30,31,32,33], ergotamine in 11 studies (range 0.3 to 51%) [14,15,16,18,20,26,28,29,31,32,33], and NSAIDs in 12 studies (range 13–85%) [14,15,16,17,19,20,21,26,27,28,29,32]. Opioids were reported in nine studies (range 1 to 37%) [14,18,19,20,21,26,29,30,31], paracetamol in six studies (range 4 to 49%) [17,19,20,29,30,32], aspirin in four studies (range 7–32%) [19,20,21,29], and non-specific analgesics in six studies (range 6 to 45%) [16,17,26,28,30,32]. The least reported pharmacological strategies were analgesic plus caffeine in two studies (range 5–31%) [17,29], and sedatives [29], steroids [26], and anti-nausea medication [26], which were each reported in one study. Finally, the use of OTC medications, in general, was reported separately and seemed to indicate that OTC medications may be widely used for managing acute migraine, with use ranging from 0.6 to 91% in four studies [15,18,22,24].

### 3.4. Types of Non-Pharmacological Strategies Reported

As shown in Table 3, the overall reported use of non-pharmacological strategies for managing acute migraine was low. Only 13 studies reported any data on non-pharmacological strategies, and few strategies were reported in each study [14,15,17,18,19,22,23,25,28,30,31,32,33]. The number of different non-pharmacological strategies reported by each study ranged from one [30] to 11 [25]. The two studies [23,25] that did not include pharmacological strategies reported the largest number of different strategies; one study reported 11 different strategies [25], and the other six different strategies [23]. Consulting a medical practitioner was reported in 10 studies, ranging between 14% and 79% [14,15,18,19,22,28,30,31,32,33]. The use of physical strategies ranged between 0.2% and 52%, where massage (studies n = 5, range 2% to 52%) [17,18,23,25,31] and acupuncture (studies n = 5, range 0.2% to 28%) [17,18,23,25,33] were most reported. Use of psychological strategies ranged between 0.4% and 68%, where relaxation (studies n = 3, range 4% to 68%) [25,31,33] and rest (studies n = 3, range 3% to 15%) [17,25,28] were the most commonly reported. Use of self-initiated strategies ranged between 0.2% and 60%, where herbal (studies n = 4, range 0.2% to 46%) [15,17,23,25], homeopathy/naturopathy (studies n = 4, range 1% to 31%) [15,18,31,33], and dietary supplements (studies n = 4, range 0.6% to 60%) [15,23,25,31] were most commonly reported.

### 3.5. Satisfaction with Management and Outcomes

Limited data were reported on outcomes and satisfaction with migraine management. Only three studies [18,21,22] identified the impact/burden of migraine. The psycho-social burden of migraines was reported as being very high in three studies. For example, Cooke et al. [18] reported 73% felt a lack of control over their lives, and 92% missed work or family activities. Migraine inhibited the ability to carry out daily activities in 79% and 17%, respectively, as stated by Ducros et al. [21] and Edmeads et al. [22]. In the three studies reporting the impact of migraine on ability to work, Cooke et al. [18] showed that the mean number of missed days per year was 20.8, Ducros et al. [21] reported a mean of 2.1 days were taken off work in the preceding three months, while Edmeads et al. showed that 11% of the reported migraine occurrences caused the headache sufferer to leave or not report to work [22].

Data for treatment efficacy and satisfaction were reported in 11 studies [14,17,18,19,20,21,26,29,30,32,33]. Four studies [14,26,30,32] indicated that satisfaction was higher when prescription medications were used than when OTC medications were used. The rate of satisfaction with medications was reported in six studies [17,18,20,21,29,33], where being very or quite satisfied ranged from 22% to 91% and being very unsatisfied ranged from 2% to 11%. Only one study, Donnet et al. [19], reported treatment was effective in 31% of respondents. Nine studies [15,16,22,23,24,25,27,28,31] reported no information on treatment outcomes and satisfaction.

### 3.6. Risk of Bias in Included Studies

The risk of bias for the 20 included studies was assessed according to six factors as described in the methods section. The risk-of-bias findings are reported in Table 4. The risk of bias was low for the purpose of the study in most studies (n = 13) as they clearly identified their purpose was to collect data on self-reported management migraine as their primary or secondary objective. The risk of bias was generally unclear for survey reliability and validity (n = 10). The risk of bias related to data collection methods was generally low (n = 18) as either telephone/in-person interviews or questionnaires were used for data collection. The risk of bias arising from the sample size was low in all studies, as no study had a sample size of less than 100. Bias due to the number of domains used to identify migraine management strategies was rated as low (n = 11), high (n = 6), unclear (n = 2) or moderate (n = 1), indicating that the types of strategies were generally restricted to medications with limited mention of other types of strategies. Conflict of interest often had a high risk of bias (n = 10) primarily due to support from pharmaceutical manufacturers for the studies.

## 4. Discussion

### 4.1. Summary of Key Findings

To our knowledge, this is the first systematic review of self-reported acute migraine management strategies among community-dwelling adults. This review included a total of 20 articles that reported pharmacological (18 studies) and non-pharmacological strategies (13 studies).

The first key finding from this study was that the most common three medications used by individuals with migraines were triptans (9–73%), ergotamine (0.3–51%), and NSAIDs (13–85%), while opioid consumption was relatively low (1–37%). The second key finding of this research was that individuals with migraines used a range of non-pharmacological strategies, which we grouped into four main domains (medical, physical, psychological, and self-initiated strategies) using the conceptual model developed by Axon et al. [13].

In terms of pharmacological strategies, this review found people used a variety of different pharmacological strategies for managing acute migraine. We found that people commonly used triptans, ergotamine, and NSAIDs. Some people used prescribed medications but did not specify which medications they were using to manage their acute migraine. Kawata et al. investigated the treatment patterns among migraine patients and revealed that patients reported high rates of acute migraine medication usage. Kawata et al. found that the most commonly reported medications were triptans (44%), acetaminophen (47%), and NSAIDs (53%) [34]. This is largely consistent with what we found among the pharmacological strategies since we found that triptans, NSAIDs, and ergotamine were the most commonly used by individuals with migraine. Another study by Antonaci et al. found that although triptans have seen increased usage since their introduction, NSAIDs continue to be used for treating acute migraines. Antonaci et al. add that NSAIDs are considered the treatment of choice for mild and moderate migraine [35]. This is consistent with what we found among the pharmacological strategies since NSAIDs were one of the most common medications used by individuals with migraines.

Regarding non-pharmacological strategies, individuals with migraine utilized medical, physical, psychological, and self-initiated non-pharmacological strategies. People usually consulted a medical practitioner about migraine, including a general physician, specialist, or visiting an emergency department. Massage, acupuncture, cold, heat, and exercise were among the physical strategies. Relaxation, yoga, rest, and psychotherapy were utilized as psychological strategies. Self-initiated strategies included doing nothing, herbs, homeopathy/naturopathy, food, and supplements. This observation is similar to a previous study on multidomain pain management strategies for chronic pain among community-dwelling people. Axon et al. [13] reported that people utilized physicians, chiropractic, and surgery among medical strategies. People utilized exercise, massage, hot/cold treatment, and acupuncture among physical strategies. Axon et al. also observed that participants utilized relaxation, prayer or meditation, counseling, and rest/sleep among psychological strategies. Self-initiated strategies included dietary or herbal supplements, diet adjustments, and complementary and alternative medicine [13]. This was consistent with what we found on how people manage their migraine. Another study by Wells et al. discussed complementary and alternative medicine use among US individuals with migraines and severe headaches. Wells et al. found that a large number of individuals with migraines or severe headaches reported using herbal or other supplements [36]. This finding aligned with our finding since we found that four studies reported the usage of dietary supplements, with range of use between 0.6% and 60%, and four studies reported the use of herbal, with a range of use between 0.2% and 46%.

### 4.2. Comparison to Clinical Guidelines

Multiple medication classes are utilized for migraines, and many clinical practice recommendations have been produced in the US and Europe. Regardless of their distinctions, all are founded on the same fundamental ideas.

The American Headache Society consensus statement categorized migraine treatments into preventive and acute treatment, and each category has its own treatment goal [37]. The goal of acute migraine treatment is rapid and constant relief from pain and accompanying symptoms without recurrence, restored ability to function, minimal re-dosing or rescue medicine requirements, self-care optimization, decreased utilization of emergency visits, and few or no adverse effects [37]. The American Headache Society recommens the use of triptans, dihydroergotamine, small-molecule calcitonin gene-related peptide (CGRP) receptor antagonists, and selective serotonin receptor agonists for moderate-to-severe migraine attacks. NSAIDs, nonopioid analgesics, acetaminophen, or caffeine-containing analgesic combinations (e.g., aspirin + acetaminophen + caffeine) are recommended for mild-to-moderate attacks [37]. Our findings showed that people mainly rely on three main pharmacological treatments, which include triptans, ergotamine, and NSAIDs. This finding aligns with the American Headache Society recommendations for mild-to-moderate migraine attacks.

Among the non-pharmacological strategies, we found that people used massage more than acupuncture (2–52% vs. 0.2–28%) for managing their migraine. The US Headache Consortium classified their recommendation based on evidence where class I is the strongest evidence and class IV is the weakest evidence. According to the US Headache Consortium, yoga has evidence grade II, while relaxation has evidence grade I [38,39]. The US headache consortium shows evidence of grade I for acupuncture and grade IV for massage. In our study, we found similar results where people used relaxation more than yoga (4–68% vs. 0.4–7%). According to the US headache consortium, using dietary supplements such as vitamin B12 has a grade I recommendation and Coenzyme Q10 has grade II [38,39]. Our study found that people used dietary supplements more than herbals (0.6–60% vs. 0.2–46%) among self-initiated strategies.

### 4.3. Implications for Patient Management

The findings of this systematic review have several implications for patient management. This includes considerations for women, access to physician care, and the role of pharmacists in managing migraine.

We found that women were more likely to participate and engage in studies related to migraine. This finding aligns with the Lay et al. report that found women of all ages, from preteens to those beyond menopause, were more likely to suffer from migraines [40]. Therefore, healthcare providers should be aware of the specific challenges that female migraine patients face when helping them manage migraines. The impact of hormonal changes, whether they are caused endogenously or exogenously, is often unexpected; as a result, considerable consideration has to be given to the several available therapy options [40]. In addition to this, regular consideration is needed towards the possibility of becoming pregnant [40]. Another point that might have influenced individuals with migraine is the accessibility of migraine patients to prescription drugs [41]. Lafata et al. compared the use of migraine preventive treatments for those with migraine and without migraine headaches and found that patients who adhered to their migraine preventive treatments were less likely to develop migraine disabilities [41]. Lafata et al. add that although the costs of preventive medications are high among those who used preventive medications, the overall treatment costs of migraine were reduced [41]. People with migraine usually do not seek medical care for their migraine headaches. Therefore, Lafata et al. suggested that programs are needed to improve diagnosis rates since it would reflect positively on migraine treatment rates [41].

Antonaci et al. suggested that effective migraine therapy depends on effective physician–patient collaboration and patient education and that the diagnosis must be clearly explained and understood from the beginning [35].

Community pharmacists have a vital role in managing migraine since they can refer patients who suffer from severe migraine to physicians, since individuals with migraine seek advice first from community pharmacists because it is more convenient for them. A community pharmacist’s role includes identifying patients in need of preventive treatment, referring them to a specialist, and ensuring that those already receiving preventive medicine are using it properly and safely, which would reduce the disease burden for all individuals with migraine [42]. Giaccone et al. investigated the community pharmacist’s role in managing headaches and found that the community pharmacist is crucial in managing patients with headaches. A qualified pharmacist may actively participate in preventative screening and therapeutic adherence monitoring [42]. This might explain the higher percentage of individuals with migraine using NSAIDs (13–85%), OTC (0.6–91%), and analgesics (6–45%) in our findings. However, Giaccone et al. added that this would not be efficient unless the community pharmacists receive adequate and continuous training on management and a reliable working relationship with the patient’s primary care physician [42]. Another study by Mehuys et al. investigated the headache characteristics and medication usage of individuals coming for self-medication with recurrent headaches. Mehuys et al. found that people with headaches misused OTC medications, such as paracetamol, NSAIDs, and combined analgesics [43]. This might explain the higher usage percentage of NSAIDs (13–85%) and paracetamol (4–49%). Mehuys et al. reported that the community pharmacist has a vital role in the teaching and referral of patients who are treating their headaches with self-medication [43].

### 4.4. Implications for Research

The findings of this systematic review have several implications for research. We have identified strategies that are less frequently used and strategies that do not have supportive evidence, which offer opportunities for future research. This includes physical strategies (massage, acupuncture, and exercise), psychological strategies, (yoga, rest, and psychotherapy), and self-initiated strategies (doing nothing, diet change, and dietary supplements). Research to establish evidence to support their use will be helpful in updating guidelines and helping optimize patient care when managing migraines.

Limited recommendations for non-pharmacological strategies could be due to the current lack of studies that focus on non-pharmacological strategies. US and European guidelines focused mainly on pharmacological management, although non-pharmacological managements have a role for individuals with migraine. Therefore, more research is needed to address the lack of guidelines for non-pharmacological strategies for managing migraine. For example, self-initiated strategies (such as doing nothing, herbal, homeopathy/naturopathy, diet, and dietary supplements) could have negative consequences that might lead to severe complications, especially if they have been used without physician supervision. For example, the Food and Drug Administration (FDA) reports serious side effects related to dietary supplements, such as itching, severe nausea, vomiting, behavioral or cognitive changes, and low blood pressure [44]. Another study by Benotsch et al. investigated the misuse of OTC products among young adults. Benotsch et al. found that the misuse of OTC drugs increased reported symptoms of depression, anxiety, and physical pain [45]. Therefore, updated guidelines are needed to ensure that people with migraines take the recommended doses of dietary supplements. Additionally, additional research is needed to see if there is a difference in migraine management between males and females. For example, it would be interesting to investigate if hormonal changes in women have an impact on migraine development. Physicians should consider women’s hormonal changes since women with hormonal imbalances might need specific treatments for their migraine [40].

### 4.5. Implications Due to COVID-19

During the coronavirus disease-19 (COVID-19) pandemic, associations between COVID-19, COVID-19 vaccination, and headache or migraine have been suggested and identified [46,47,48]. For instance, a narrative review discusses the innate immune response to viral infection, which may be linked to headache [46]. In addition, a case series has identified a link between visual aura associated with migraine and COVID-19 infection [47]. Finally, a recent systematic review and meta-analysis identified a two-fold increase in developing headache after a vaccine [48]. There is scope for additional research to investigate the influence of COVID-19 on migraine prevalence and management.

### 4.6. Study Limitations

In addition to the methodological strengths and weaknesses of the studies included, there are a few limitations to this systematic review. The scope of the terms ‘self-management’ and ‘self-reported’ in the literature and the large heterogeneity in terminology has repeatedly been highlighted in previous systematic reviews and meta-analyses [49,50]. Heterogeneity was also present from the broad eligibility criteria that included different study designs, different populations, and studies from different countries. However, this did allow the review to be more comprehensive than it otherwise would have been. Subtle variations in self-management/self-reported definitions can result in substantial differences in selected studies. The exclusion criteria used in this review was preventive migraine management; however, many studies did not clearly describe the nature of migraine. This review defined migraine according to the International Classification of Headache Disorders third edition (ICHD-3) criteria [3]. However, it is unclear if all studies in this review used the same definition. It is likely not the case, as several studies included were published before the ICHD-3 criteria were released. Data on the characteristics of migraine were not captured, as this was beyond the scope of capturing data on migraine management strategies and was not always available. Few studies included in this review did not specify the strength and subtype of pharmacological medications used by migraineurs. The methodological quality domains of the included studies were unclear, with not enough information on the validity and reliability of the surveys used in the questionnaire studies. There was also substantial bias in terms of conflict of interest.

## 5. Conclusions

The primary management strategy for migraine was the use of triptans, ergotamine, and NSAID, while the use of opioids was not that high, both consistent with practice guidelines for the management of migraine. Reported use of non-pharmacological management strategies was limited, indicating that more study is needed to establish high-quality evidence for the effective use of non-pharmacological strategies.

## Figures and Tables

**Table 1:** Characteristics of studies included in the systematic review.

Author, Year	Country	Purpose	Total N	Female %	Mean (SD) Age	Source Population	Survey Type
Adelman, 2000 [14]	USA	Experiences and current treatment practices	801	N/A	N/A	General population with severe headache	Tel
Brusa, 2014 [15]	Italy	Recommendations by pharmacist to manage patient’s migraine and common analgesics medications used	1042	82	44.1 (13.5)	Pharmacy customers with headache	SAQ
Brusa, 2019 [16]	Italy	Distribution of migraine headaches and overuse of medicines among people seeking medication in pharmacies	4424	45	45.1	Pharmacy customers seeking headache medications	PQ
Chang, 2021 [17]	Taiwan	Effectiveness of pain management strategies; relationship between number of strategies and effectiveness	174	N/A	38.5 (11.8)	Adults age 20–65	FFI
Cooke, 2010 [18]	Canada	Prevalence of migraine in Canadian women and treatment practices and psychological burden	300	100	N/A	Adults age > 18	Tel
Donnet, 2009 [19]	France	Migraine management among pharmacy personnel who were migraineurs	2094	90	N/A	Pharmacy personnel	SAQ
Donnet, 2010 [20]	France	Perceptions of migraine among neurologists and treatments used for their own migraines	179	37	47.7 (9.8)	Community neurologists	SAQ
Ducros, 2011 [21]	France	Headache treatment patterns in general practitioners who suffered from migraine themselves	277	23	50.1 (7.1)	General practitioners	SAQ
Edmeads, 1993 [22]	Canada	Prevalence and effects of migraine on lifestyles, consulting behavior and medication use	138	N/A	N/A	Adults age > 15	Tel
Ertem, 2019 [23]	Turkey	Association between complementary and alternative usage and chronicity of migraine	100	82	42.8 (11.4)	Adults age > 18	PQ
Hirata, 2021 [24]	Japan	Provide up-to-date assessment of migraine epidemiology in Japan	17,071	66.5	40.7 (13.0)	Adults age > 18	OS
Ismail, 2021 [25]	Kuwait	Assess traditional medicine in migraine treatment during COVID-19	1018	86.9	34 (9.5)	Headache clinic patients	OS
Jelinski, 2006 [26]	Canada	Clinical features and pharmacological treatment of migraine patients	606	83	39.7 (12.9)	Headache outpatient database	RD
Landy, 2012 [27]	USA	Interrelationship of migraine onset, duration and time to treatment	509	75	41.0 (10.0)	Chronic illness panel	SAQ
Lebedeva, 2017 [28]	Russia	Evidence-based diagnosis and treatment of headache disorders	484	43	31.7	Adults age 18–65	FFI
Lipton, 2018 [29]	USA	Assess gender difference in sociodemographic and headache features, consultation and diagnosis patterns, and treatment patterns	15,133	73	43.1 (13.6)	Adults age > 18	OS
Lucas, 2006 [30]	France	Proportion of migraineurs who are self-aware of their disease	1652	68	41.2 (14.5)	Adults age > 18	SAQ
Peters, 2005 [31]	UK	Headache management over the last 12 months	356	90	49.1 (9.3)	Adults age 18–65	SAQ
Viticchi, 2018 [32]	Italy	Disease awareness, general approach, and impact on working activity	294	80	42.1 (10.6)	Adults age > 18	SAQ
Vukovic, 2010 [33]	Croatia	Treatment patterns of migraine	289	70	41.0 (14.0)	Adults age > 18	SAQ

SD = standard deviation; N/A = not applicable; COVID-19 = coronavirus disease 2019; Tel = telephone survey/interview; SAQ = self-administered questionnaire; PQ = paper-based questionnaire; FFI = face-to-face interview; OS = online survey; RD = retrospective database.

**Table 2:** Percentage of each type of pharmacological pain management strategy reported by individuals with migraine.

Study	Trip	Ergo	Opioid	NSAID	Para	Asp	Analg	An-caf	Sed	Ste	A/nau	Other
	%	%	%	%	%	%	%	%	%	%	%	%
Adelman [14]		4	37	21								
Brusa [15]	43	10		45								OTC: 0.6
Brusa [16]	57	51		48			39					
Chang [17]				13	21		13	5				
Cooke [18]	8	1	23									OTC: 38
Donnet [19]	32		11	37	10	7						
Donnet [20]	50	3	4	57	27	32						2
Ducros [21]	73		9	85		16						4
Edmeads [22]												OTC: 91 Rx: 41
Hirata [24]	20											OTC: 80
Jelinski [26]	49	3	1	59			24			0.5	8	
Landy [27]	59			41								
Lebedeva [28]	6	2		44			45					
Lipton [29]	17	1	11	69	36	13		31	4			
Lucas [30]	23		7		49		15					
Peters [31]	58	8	3									
Viticchi [32]	9	0.3		17	4		6					
Vukovic [33]	36	22										
Range	9–73	0.3–51	1–37	13–85	4–49	7–32	6–45	5–31	4	0.5	8	OTC: 0.6–91

Trip = triptan; Ergo = ergotamine; NSAID = non-steroidal anti-inflammatory drugs; Para = paracetamol; Asp = aspirin; Analg = unspecified analgesics; An-caf = analgesic with caffeine; Sed = sedatives; Ste = steroids; A/nau = anti-nausea; OTC = over the counter medications; Rx = prescription medications. Two studies [23,25] did not report any pharmacological pain management strategies and were therefore not included in this table.

**Table 3:** Percentages for each type of non-pharmacological pain management strategy reported by individuals with migraine.

	Medical	Physical					Psychological				Self-Initiated				
	Cons. Med	Mas	Acu	Col	Hot	Exe	Rel	Yog	Rest	Psy	No	Her	H/N	Diet	DS
	%	%	%	%	%	%	%	%	%	%	%	%	%	%	%
Adelman [14]	GP:56										11				
Brusa [15]	GP:40, SP:22											0.2	2		0.6
Chang [17]		11	7						6			11			
Cooke [18]	GP:62, SP:50	2	1								10		1		
Donnet [19]	GP:38														
Edmeads [22]	ED:14														
Ertem [23]		48	28			12				14		24			60
Ismail [25]		52	0.2	23	8	13	17	0.4	15			46		20	22
Lebedeva [28]	GP:40, SP:60								3		44				
Lucas [30]	GP/SP:60														
Peters [31]	GP:79	47		46	36		68						31		19
Viticchi [32]	GP/SP:51														
Vukovic [33]	GP:64		9				4	7					1		
Ranges	14–79	2–52	0.2–28	23–46	8–36	12–13	4–68	0.4–7	3–15	14	10–44	0.2–46	1–31	20	0.6–60

Cons. Med = consult medical practitioner; GP = general physician; SP = specialist physician, usually a neurologist; ED = emergency department; Mas = massage; Acu = acupuncture; Col = cold; Exe = exercise; Rel = relaxation; Yog = yoga; Psy = psychotherapy; No = nothing; Her = herbals; H/N = homeopathy/naturopathy; DS = dietary supplements. Note: Several of the non-pharmacological strategies listed are not usually considered treatments for acute migraine; however, these are the strategies identified by the respondents who may or may not be using strategies according to how they are officially categorized. Seven studies [16,20,21,24,26,27,29] did not report any pharmacological pain management strategies and were therefore not included in this table.

**Table 4:** Risk of bias assessment for included studies.

Study	Purpose	Survey Reliability and Validity	Data Collection Method	Sample Size	Categories Used to Identify Strategies	Conflict of Interest
Adelman [14]	High	Unclear	Unclear	Low	High	High
Brusa [15]	Unclear	Unclear	Low	Low	High	Unclear
Brusa [16]	Unclear	Low	Low	Low	Low	Low
Chang [17]	Low	Low	low	Low	Low	Low
Cooke [18]	Low	Low	Low	Low	Low	High
Donnet [19]	Unclear	Unclear	Low	Low	High	High
Donnet [20]	Low	Unclear	Low	Low	Low	High
Ducros [21]	Low	Unclear	Low	Low	High	High
Edmeads [22]	Low	Unclear	Low	Low	Unclear	High
Ertem [23]	Low	Moderate	Low	Low	Low	Low
Hirata [24]	Low	Low	Low	Low	Low	Low
Ismail [25]	Low	Moderate	Low	Low	Moderate	Low
Jelinski [26]	Low	Unclear	Unclear	Low	Low	High
Landy [27]	High	Unclear	Low	Low	High	High
Lebedeva [28]	Low	Low	Low	Low	Low	Low
Lipton [29]	Low	Low	Low	Low	Low	Moderate
Lucas [30]	High	Low	Low	Low	Unclear	High
Peters [31]	Low	Low	Low	Low	Low	High
Viticchi [32]	Unclear	Unclear	Low	Low	High	Low
Vukovic [33]	Low	Unclear	Low	Low	Low	Unclear

Ratings such as low, unclear, and high were used to assess the risk of bias based on a tool developed specifically for this study purpose. Low could indicate a low risk of bias for that domain, while unclear is when bias could not be clearly estimated or seen, while high means a great risk of bias seen for that domain assessed.

## Data Availability

Available upon request from the corresponding author.

## Appendix A

PubMed/Medline Search Strategy.

(((“Migraine Disorders” [MESH] OR “Migraine without aura” [MESH] OR “Migraine with aura” [MESH] OR “migraineurs” [MESH] OR “Migraine” [ALL] OR “Familial Migraine” [ALL] OR “migrainous headache” [ALL] OR “hemicrania” [ALL] OR “Migraine disorders” [ALL] OR “Status hemicranicus” [ALL] OR “Migraine aura” [ALL] OR “Migrainous aura” [ALL] OR “Classic migraine” [ALL] OR “Migraine with aura” [ALL] OR “Common migraine” [ALL] OR “migraine without aura” [ALL]) AND (“self-report” [MESH] OR “diagnostic self evaluation” [MESH] OR “surveys and questionnaires” [MESH] OR “self-assessment” [MESH] OR “self medication” [MESH] OR “personal narratives” [Publication Type] OR “self-concept” [MESH] OR “self-disclosure” [MESH] OR “self care” [MESH] OR “patient participation” [MESH] OR “self-management” [MESH] OR “self-treatment” [ALL] OR “patient diary” [ALL] OR “patient journal” [ALL] OR “personal assessment” [ALL] OR “personal diary” [ALL] OR “personal evaluation” [ALL] OR “personal monitoring” [ALL] OR “personal narrative” [ALL] OR “personal recollection” [ALL] OR “personal report” [ALL] OR “self analysis” [ALL] OR “self-analysis” [ALL] OR “self assessment” [ALL] OR “self-assessment” [ALL] OR “self evaluation” [ALL] OR “self-evaluation” [ALL] OR “self-concept” [ALL] OR “self concept” [ALL] OR “self monitoring” [ALL] OR “self-monitoring” [ALL] OR “self-disclosure” [ALL] OR “self narrative” [ALL] OR “self-narrative” [ALL] OR “self observation” [ALL] OR “self-observation” [ALL] OR “self report” [ALL] OR “self-report” [ALL] OR “self-administered questionnaire” [ALL] OR “self administered questionnaire” [ALL] OR “surveys and questionnaires” [ALL] OR “diagnostic self evaluation” [ALL] OR “diagnostic self-evaluation” [ALL] OR “self medication” [ALL] OR “self-mediation” [ALL] OR “health surveys” [ALL] OR “self care” [ALL] OR “self-care” [ALL] OR “self help” [ALL] OR “self-help” [ALL] OR “patient participation” [ALL] OR “self management” [ALL] OR “self-management” [ALL])) AND (“Adult” [MESH] OR “community participation/methods” [MESH] OR “Community dwelling adult*” [ALL] OR “Community adult*” [ALL] OR “Community” [ALL] OR “Community population” [ALL] OR “Adult” [ALL] OR “Adults” [ALL] OR “Grownup” [ALL] OR “Grown-up*” [ALL] OR “Grownup*” [ALL] OR “population based” [ALL] OR “population-based” [ALL])) AND ((“cross-sectional studies” [MESH] OR “prevalence” [MESH] OR “observational study” [Publication Type] OR “health surveys” [MESH] OR “healthcare survey” [MESH] OR “longitudinal studies” [MESH] OR “qualitative research” [MESH] OR “focus groups” [MESH] OR “interview” [Publication Type] OR “cross-sectional studies” [ALL] OR “prevalence” [ALL] OR “observational study” [ALL] OR “health surveys” [ALL] OR “longitudinal studies” [ALL])).
